# Exploring the Enigmatic Spread and Spatial Dynamics of *Bursatella leachii* in the Mediterranean Sea

**DOI:** 10.3390/biology14020133

**Published:** 2025-01-27

**Authors:** Luca Castriota, Manuela Falautano, Teresa Maggio, Patrizia Perzia

**Affiliations:** Unit for Conservation Management and Sustainable Use of Fish and Marine Resources, Italian Institute for Environmental Protection and Research, Department for the Monitoring and Protection of the Environment and for the Conservation of Biodiversity, Lungomare Cristoforo Colombo 4521 (Ex Complesso Roosevelt), Località Addaura, 90149 Palermo, Italy; luca.castriota@isprambiente.it (L.C.); manuela.falautano@isprambiente.it (M.F.); teresa.maggio@isprambiente.it (T.M.)

**Keywords:** alien species, cryptogenic species, invasive species, spatio-temporal indicators, invasion pattern, invasion hotspots, spatio-temporal statistics

## Abstract

The invasion history of the gastropod mollusk *Bursatella leachii* in the Mediterranean Sea demonstrates a dynamic progression through distinct phases, comprising arrival, establishment, and expansion phases. Initial records in the 1930s trace its entry through the Suez Canal, likely followed by larval transport to the Aegean Sea facilitated by natural currents. The subsequent spread to Maltese and Italian waters suggests secondary dispersion favored by maritime traffic or aquaculture activities. The establishment phase shows a significant increase in occurrences and spatial dispersion, with aggregated nuclei in the central Mediterranean. The expansion phase was characterized by rapid dispersal and intensified densities, particularly in the Aegean, Adriatic, and Spanish coasts. These findings underscore the species’ capability to invade from multiple fronts, driven by both natural and human-mediated processes. A new location in the Strait of Sicily, i.e., the island of Lampedusa, is reported in the present paper for the species. Given the low number of observations, its establishment with a viable population on the island is not confirmed, although its detection validates the Straits of Sicily as a key area of intense settlement of the species.

## Simple Summary

The invasion of the gastropod *Bursatella leachii* in the Mediterranean Sea occurred in three main phases, namely arrival, establishment, and expansion. The species first entered the Mediterranean in the 1930s via the Suez Canal. It likely reached the Aegean Sea via larval transport facilitated by natural currents. Its subsequent spread to Maltese and Italian waters points to secondary dispersion, possibly aided by maritime traffic or aquaculture activities. During the establishment phase, sightings of *B. leachii* increased significantly, with clusters forming in the central Mediterranean. The expansion phase exhibits a rapid spread and higher population densities, especially along the Aegean, Adriatic, and Spanish coasts. These findings highlight the species’ ability to invade new areas driven by a combination of natural dispersal and human activities.

## 1. Introduction

One of the most stimulating challenges for bioinvasion researchers is to discover the origin of the invading organisms as well as the factors promoting their invasion to be able to manage the phenomenon should it prove harmful to the environment and its inhabitants. The Mediterranean is among the most invaded seas in the world, counting over a thousand non-indigenous species introduced through human activities [1], many of which are considered established and a few invasive [2]. (Established non-indigenous species are species who naturally reproduce in the recipient areas maintaining a stable population. Invasive alien species are a subset of established non-indigenous species, which rapidly spread and/or have a negative impact on biological diversity. See Zenetos et al. [3] for details). In addition, species enter the Mediterranean through natural routes like the Strait of Gibraltar, a process further facilitated by seawater warming, at least as far as tropical and subtropical Atlantic species are concerned [4]. To understand the ecological processes underlying a species’ invasion, it is essential to examine its invasion history, starting with its origin, potential introduction vectors, and diffusion pathways. However, identifying the routes of origin of invading species is not always possible, since they may manifest invasive characteristics (e.g., high reproductive rate, rapid spread in new areas, ability to survive in highly variable environments, high competitive ability) even several years after their establishment.

When there is only one entry site of the species and its distribution records follow a clear spatial and temporal progression, it is possible to identify the invasion pathway. This scenario is particularly evident in several Lessepsian species—species native to the Indo-Pacific and Indian oceans entering the Mediterranean via the Suez Canal, e.g., the fishes *Fistularia commersonii* and *Pterois miles*. These species typically show a colonization pattern extending from the Levant Basin toward the western Mediterranean Basin [5,6], as well as in some ranges—expanding Atlantic species colonizing in the opposite direction (e.g., the fishes *Sphoeroides pachygaster* and *Seriola fasciata*) [7,8]. Several other alien species exhibit more complex distribution patterns, which does not allow for the immediate identification of a colonization path. This is the case of the ragged sea hare *Bursatella leachii* (de Blainville, 1817), a circumtropical tectipleuran gastropod with a nearly worldwide distribution in warm temperate-to-tropical waters. It commonly lives on sandy or muddy bottoms in intertidal and subtidal zones of sheltered bays and estuarine habitats and can also be found in aquaculture ponds and harbors [9,10,11,12,13]. *B. leachii* is a benthic species characterized by planktotrophic larval development, rapid larval and postlarval growth, and the ability to delay metamorphosis to allow the larva to travel long distances and choose the substrate suitable for the development of the adult stage [14]. The distribution of *B. leachii* seems related to the presence on the substrate of its favorite food that is made up of microalgae and macroalgae [14,15,16]. A massive aggregation of individuals can be observed in certain periods, probably linked to optimal hydrological conditions [16].

The species entered the Mediterranean Sea around the 1930s, probably via the Suez Canal and since then, it has been recorded in different Mediterranean regions, recently reaching its westernmost range in Morocco [10]. Although its origin was considered to be the Red Sea, recent molecular analyses have disproved this, highlighting that Mediterranean and Atlantic specimens share similar or identical haplotypes, slightly different from the Indo-Pacific ones [17]. The authors analyzed specimens from different locations through the mitochondrial COI gene, a molecular marker commonly used to study the phylogeography and invasion history of a species. The analysis of the COI sequences from different specimens indeed allows us to reconstruct the invasion history of the species through a connection network built with the different nucleotide sequences (haplotypes). In the case of *B. leachii*, COI sequences from the Levant basin and the Red Sea were not considered in the network, while they would have been necessary to properly explain the relationships among different haplotypes given that the species is widely distributed in the Atlantic Ocean as well as in the Indo-Pacific region, including the Red Sea.

As a result, the question of the invasion pathway of Mediterranean *B. leachii* remains unresolved, leading part of the scientific community to consider the species cryptogenic (i.e., of unknown origin) in the Mediterranean [1].

Geostatistical analyses and spatial indicators could be highly useful tools to enhance our understanding of the dispersal dynamics of this species, as they provide valuable insights into aggregation trends, spatial structure, and key distribution characteristics [7,18]. These metrics enable the quantification of how occurrences are distributed across time and space, facilitating the identification of patterns such as clustering, dispersion, and aggregation nuclei. Such patterns are crucial for understanding the mechanisms driving species distribution [7,8,18,19,20,21].

This study aimed to (1) update the species’ distribution within the Mediterranean Sea, including a new locality in the Strait of Sicily; (2) analyze its distribution, aggregation patterns, and spatial structure in the Mediterranean Sea; (3) investigate the spatio-temporal dynamics of its occurrences; and (4) identify persistent hotspots useful for monitoring and management strategies.

## 2. Materials and Methods

The study of the distribution of *Bursatella leachii* in the Mediterranean Sea and surrounding areas was carried out starting from the database provided by [22], to which records obtained from the literature up to April 2024 were added. Studies were identified using Google (google.com), Google Scholar (scholar.google.com), and ResearchGate (researchgate.net) search engines (accessed up to April 2024), as well as consulting the online archives of the main scientific journals that publish studies on the topic of bioinvasions. Citizen science archives (e.g., GBIF.org) were also consulted. Several combinations of keywords were used to identify the relevant literature, such as the following: “Bursatella leachii”, the synonym “Notarchus leachii”, “Bursatella”, “Notarchus”, and “ragged sea hare”, as well as each keyword alone or combined with Mediterranean, Suez, or Red Sea. The reference lists of the publications found were also used as bibliographic sources. The data warehouse included the year of record (if missing, the year of publication was used), location, country, geographic coordinates, and source. Doubtful or duplicate information was excluded to ensure data reliability.

### Bursatella leachii Distribution, Aggregation Patterns, and Spatial Structure in Mediterranean Sea

The spatial data were processed using ArcMap 10.3 (ESRI, Redlands, CA, USA), considering only the first recorded occurrence (in chronological order) within each 0.05° lat/long grid to minimize the effects of preferential sampling [7]. This sub-dataset was used for all analyses, regardless of the number of specimens recorded.

The invasion phases of *B. leachii* were identified from the cumulative curve of occurrences, which was divided into time intervals based on the most prominent changes in slope, according to Perzia et al. [18]. The least squares method was applied to determine the regression line equations for each interval, allowing for an evaluation of the rate of increase in occurrences over time. The y-axis values also represent the expansion areas, defined by the cumulative number of 0.05° lat/long grid cells affected by occurrences.

To characterize the distribution, aggregation patterns, and spatial structure of *B. leachii* occurrences in the Mediterranean Sea, qualitative and quantitative multi-parameter modeling was conducted using the spatial analysis and spatial statistics toolboxes in ArcMap.

The Incremental Spatial Autocorrelation tool, based on Global Moran’s Index, was used to detect and measure spatial clustering or dispersion of *B. leachii* occurrences across varying distances. Statistically significant peaks in z-scores indicate key distances where spatial patterns are most pronounced [23,24]. The first peak distance was used to define the appropriate scale for kernel density analysis (distance radius) and hotspot/outlier detection (distance band), whose methods are described below.

Kernel density analysis is a statistical method widely used to identify spatial patterns, trends, or clusters in species occurrence data [8,18,20,21]. The output is a continuous surface representing density, where higher values indicate clusters and lower values reflect species dispersion [25]. Cumulative kernel density analyses were performed for different time periods corresponding to the invasion phases of *B. leachii* to (i) map occurrence density, (ii) compare density surfaces over time, and (iii) identify areas of expansion and persistent high-density occurrences.

The Getis–Ord Gi* (GOG*) statistic is a local spatial analysis method used to identify statistically significant clusters of high (hotspots) or low (cold spots) values within a dataset, often applied in ecological studies [8,18,20,21]. It generates z-scores and *p*-values for each spatial feature based on its value, neighboring values, and a distance-based weighting function [26]. Using years of occurrences as the feature’s value, positive z-scores indicate recent formations (hotspots), negative z-scores indicate older formations (cold spots), and not significant z-scores suggest random patterns. For *B. leachii*, this analysis revealed (i) initial and current spread directions and (ii) areas of dispersion and settlement. The Anselin local Moran’s I (AMI) method was also used to detect spatial outliers, identifying records significantly different from their neighbors [27]. High outliers represent recent records surrounded by older ones, while low outliers indicate older records among more recent ones.

The key characteristics of distribution—central tendency, directional dispersion, and directional trends—were analyzed across different time periods to understand the temporal and spatial evolution of the species’ distribution [8,18,20,21]. These analyses track shifts in the center of gravity (median center), measure the extent of spread (directional dispersion), and determine the predominant directional shifts (directional trends) [28,29]. Additionally, changes in the shape of the distribution (e.g., more dispersed, compact, or elongated) were examined to identify species’ spatial patterns of expansion or contraction over time.

Table 1 summarizes the analyses and spatial–temporal indicators utilized in this study, along with the tools, spatial and temporal scales, and their ecological significance.

## 3. Results

### 3.1. Invasion History and Spatial–Temporal Patterns of B. leachii Distribution in the Mediterranean Sea

A total of 525 occurrences of *B. leachii* were recorded along the coasts of 21 Mediterranean countries and just inside the Suez Canal (Mediterranean side), 89 of which were drawn from the GBIF database [30]; the dataset selected for analysis includes 255 records.

**Figure 1:**
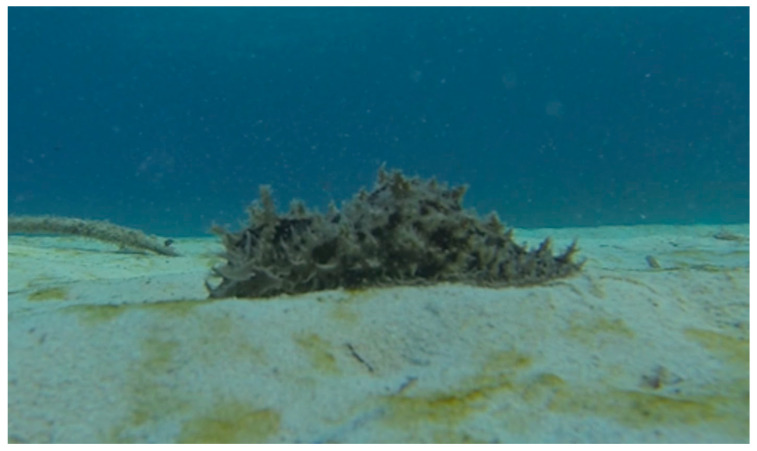
Specimen of *B. leachii* spotted in autumn 2017 in Lampedusa Island.

During a local ecological knowledge activity carried out in Lampedusa Island (Sicily) [31], we were able to ascertain the presence of *B. leachii* along the coasts of the island, as documented by a video from which the still image in Figure 1 was extracted. An individual of this species was spotted and filmed by diving guides in autumn 2017 on a sandy substrate in shallow water in the southeast coast. However, according to other diving guides, the species has been present and abundant in this area since 2009, although no video or photographic documentation is available to prove it.

**Figure 2:**
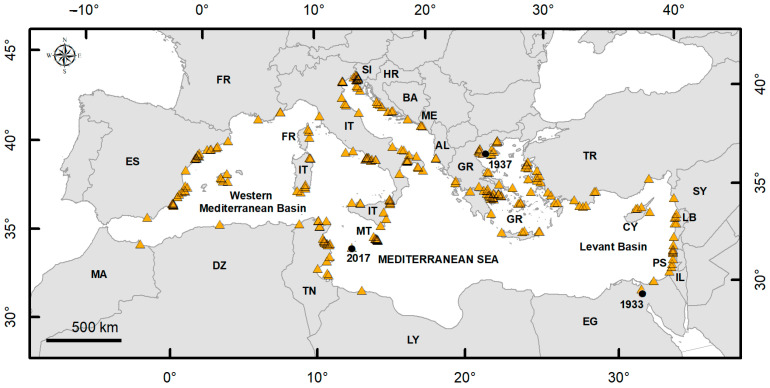
Overall distribution of *B. leachii* in the Mediterranean Sea (yellow triangles). The black circles indicate the first record in the Suez Canal in 1933, the first record in the Mediterranean Sea in 1937, and the new occurrence in 2017 documented in the present paper. ISO 3166 country codes were used to indicate the countries in which *B. leachii* occurs.

Figure 2 illustrates the overall distribution of the species in the Mediterranean Sea, with the first record in the Suez Canal in 1933 and the first Mediterranean record in Greece in 1937 highlighted. The map also includes a new locality for *B. leachii* documented in this paper.

**Figure 3:**
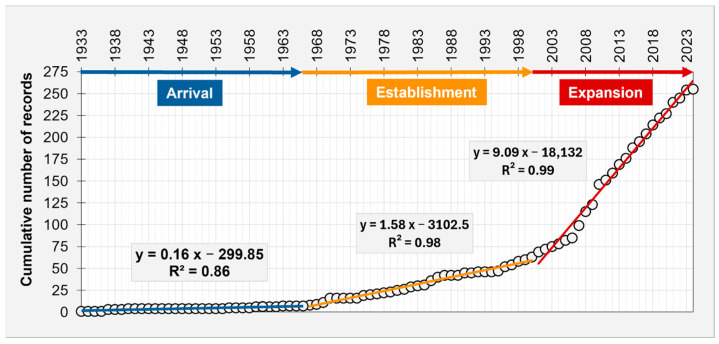
Cumulative curve of *B. leachii* occurrences in the Mediterranean Sea (circles) showing the three phases of the invasion process, namely arrival (1933–1966), establishment (1967–2000), and expansion (2001–2024) represented by blue, yellow, and red arrows, respectively. The equations of the three regression lines with the correspondent R^2^ are also reported. Only the first records within a 0.05° lat/long grid were considered for analysis. The cumulative number of records also corresponds to the cumulative number of grid cells affected by the occurrences over time.

Figure 3 shows the cumulative curve of occurrences corresponding to the cumulative number of cells in the grid affected by the presence of the species over time. Three slope changes were evident, attributable to species’ phases of invasion in the Mediterranean Sea, with arrival from 1933 to 1966; establishment from 1967 to 2000; and expansion from 2001 to 2024.

Figure 3 also reports the equations of the regression lines together with the corresponding R^2^ values.

The positive variations in slope indicate an increasing rate of occurrences and an expansion in the number of grid cells over time. The cumulative curve showed a plateau phase lasting about 33 years (arrival phase), during which only eight records were documented (slope = 0.16 ± 0.01). This period was followed by an establishment phase of about 33 years, marked by a gradual increase in the number of records (55 occurrences). The invasion of *B. leachii* accelerated significantly in the last twenty-three years (slope = 9.09 ± 0.21) compared to the previous sixty-eight years.

**Figure 4:**
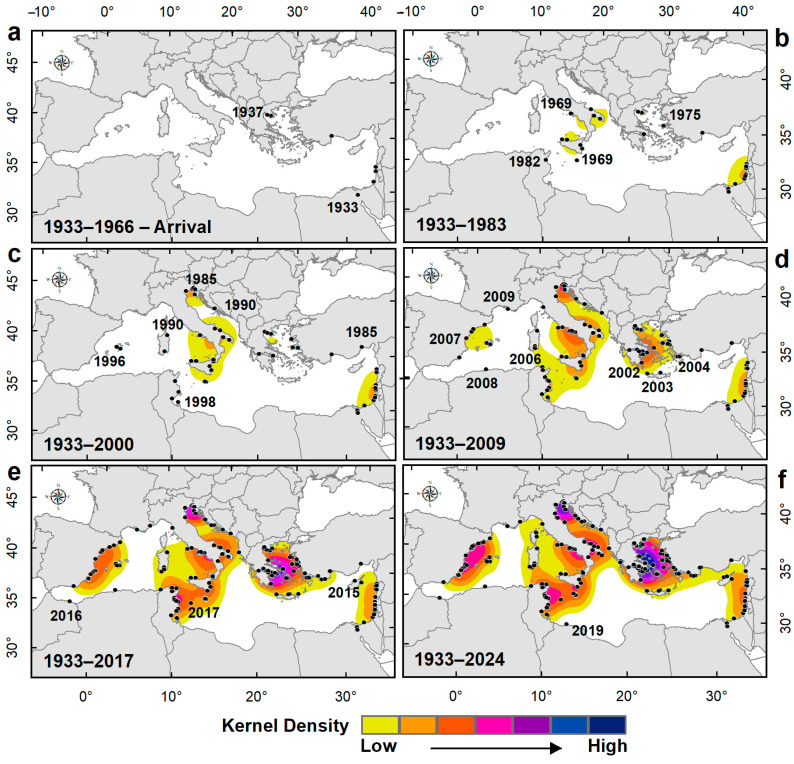
Period-to-period cumulative variation in space and time of *B. leachii* occurrence in the Mediterranean Sea together with the kernel density maps. (**a**) The 1933–1966 period, corresponding to the arrival phase of *B. leachii*. (**b**,**c**) The 1933–1983 and 1933–2000 periods, comprising the establishment phase. (**d**–**f**) The 1933–2009, 1933–2017, and 1933–2024 periods, comprising the expansion phase. The black circles indicate the records of *B. leachii* in the Mediterranean Sea.

Figure 4a–f illustrates the results of the density hotspot analysis. The kernel density maps show period-to-period variation in both space and time, with high-density areas of occurrences in the Aegean Sea and northern Adriatic (highlighted in blue in Figure 4f). During the first period, from 1933 to 1966, very few records were documented, with one in the Suez Canal and seven in the Mediterranean Sea (specifically in the Levantine Basin and Greece) (Figure 4a). This period was followed by an establishment phase lasting about 33 years, during which the species simultaneously colonized the coasts of Israel and southern Italy (Figure 4b) and, subsequently, the northern Adriatic Sea (Figure 4c). The number of records gradually increased, leading to the first aggregation nucleus of *B. leachii* records in these areas. In the third phase, the Sicilian aggregation nucleus extended toward the Strait of Sicily and the southern Tyrrhenian Sea (Figure 4d), becoming increasingly evident since 2010 (Figure 4e). A new aggregation nucleus appeared along the Spanish Mediterranean coasts, while the Aegean one intensified. The density of these nuclei reinforced over time by additional records in nearby areas, evolving into a persistent area (Figure 4d–f).

### 3.2. Aggregation Patterns and Spatial Structure

**Figure 5:**
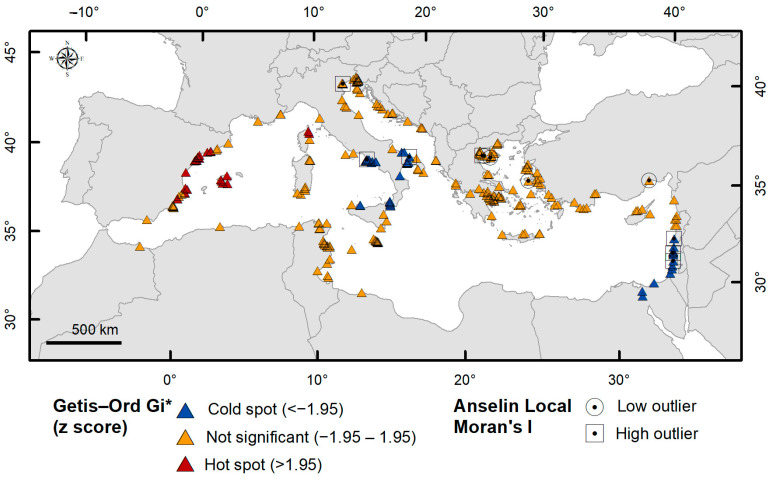
Results of the hot spot (Getis–Ord Gi*), and outlier analysis (Anselin local Moran’s I) on records of *B. leachii* in the Mediterranean Sea. Areas with statistically significant spatial clustering (cold spot—blue triangle; hot spot—red triangles) were identified. Low outliers (ring) and high outliers (square) were detected. Yellow triangles indicate records with non-significant index values.

The distribution of *B. leachii* shows a weak spatial autocorrelation at the global scale (expected GMI index = −0.004; GMI = 0.19; z > 2.58; *p* < 0.01), suggesting a non-random change in the spatial aggregation pattern over time. At a local scale, the GOG* analysis (Figure 5) revealed two areas with statistically significant cold spots (z < −1.95) in the Levantine Basin and southern Italy, corresponding to the species’ initial spread direction. Hot spots (z > 1.95) were detected in the western Mediterranean Basin along the Spanish coast. Occurrences in the Aegean, Adriatic, and Strait of Sicily had non-significant index values. High and low spatial outliers were also reported in Figure 5.

### 3.3. Key Characteristics of Distribution

**Figure 6:**
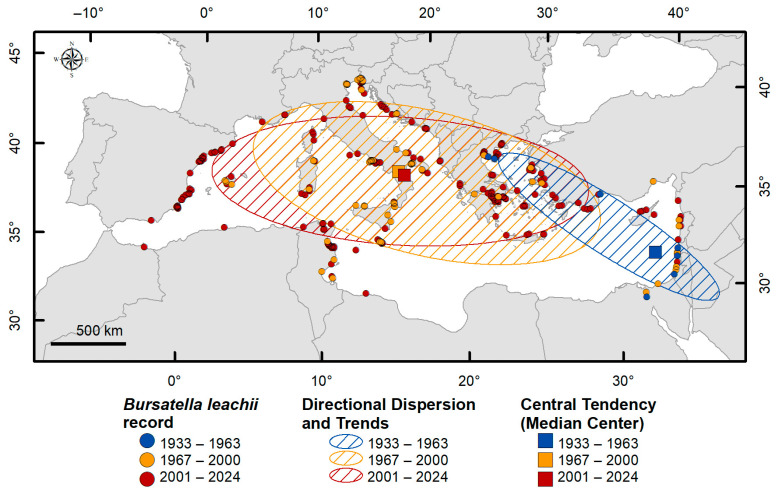
Central tendency (as median center), directional dispersion, and directional trends of *B. leachii* distribution in the Mediterranean Sea, calculated for the periods 1933–1963, 1967–2000, and 2001–2024.

Table 2 and Figure 6 outline the key distribution characteristics of *B. leachii* in the Mediterranean Sea, including central tendency (median center), directional dispersion, and directional trend, calculated for the periods 1933–1963, 1967–2000, and 2001–2024.

Comparing the size, shape, and overlap of the ellipses indicates that the distribution shape of *B. leachii* changes over space and time, especially between the first period and the subsequent ones. During the first period (arrival phase), the central tendency was in the Levantine Basin. The directional dispersion and trend extended from the Levantine coast to the Aegean Sea, forming a very elongated and narrow ellipse. From 1967 to 2000, the median center shifted further northwest, toward the central Mediterranean Sea. Spatial dispersion showed a significant change in shape and direction compared to the first period, indicating a higher dispersion of records than the previous period, with XStdDist increasing from 866 to 1184 km and YStdDist from 182 to 479 km. Distribution key characteristics in the third period are similar to the second one, where central tendencies are very close to each other and the ellipses partially overlap. During this period, YStdDist dispersion increases slightly while XStdDist decreases.

## 4. Discussion

*Bursatella leachii* has been classified under different names in the past, sometimes having been ascribed under different genera; at other times, various subspecies have been attributed to it on the basis of characters later recognized as not sufficient. A thorough revision of the taxonomic issue has been addressed by Bazzicalupo et al. [32], who found no consistent genetic differences between specimens belonging to the different subspecies previously assigned. To the list of synonyms reported by the authors must also be added *Notarchus savignyi* Audouin, reported in high abundance by Gruvel [33] in 1933–1934 in some lagoons of Lake Timsah, associated with *Halophila* meadows. This represents the first record of the species in the Suez Canal. This record has often gone unnoticed in the literature, perhaps due to the different name given to the species. As a result, the first record of *B. leachii* in the Suez Canal was considered to be that by Barash and Danin [34], who reported the species further north in the Canal in December 1967.

Directional trends indicate an initial confinement to the Levantine and Aegean Seas, transitioning to broader Mediterranean dominance over time. The non-random spatial distribution and increasing dispersion distances from 1933 to 2024 underscore the influence of changing environmental and human-mediated conditions in driving *B. leachii*’s success. Interestingly, much of the southeastern Mediterranean (particularly along the coasts of Libya and Egypt) appears not to be colonized by *B. leachii*. This situation is also observed in the distribution of other invasive species with different ecological features [8,21] and is probably due to the reduced research effort, which has only recently increased in these areas, rather than to the lack of suitable environments for the colonization of the species. The increased participation of citizens in reporting alien species, as well as the increased interest in the topic, will probably fill this gap in the future.

According to our results, the invasion history and spatial–temporal patterns of *B. leachii* in the Mediterranean Sea reflected a dynamic progression through distinct invasion phases, namely arrival, establishment, and expansion. The cumulative curve and spatial density analyses highlighted the species’ ability to adapt and proliferate across diverse Mediterranean regions. The low number of records detected in the initial period of the invasion could be related to the lower intensity of research and the reduced presence of observers (i.e., divers) of that period. Although this may represent a bias in the study of bioinvasions, as described in Perzia et al. [18], the trend obtained for *B. leachii* follows the general pattern of bioinvasions [35], as well as that of another invasive species detected in the Mediterranean in more recent times [18].

The species exhibited a slow initial spread (1933–1966) in the Levantine Basin, i.e., a lag time corresponding to the arrival phase, with limited dispersal capacity. From the literature data, it emerged that the first records of this species in the Mediterranean date back to 1937 in the northern Aegean [36] and, in the years around this date (estimated before or in 1940), along the eastern Levantine coast [37]. According to the central tendency of the distribution, this latter area represents the starting point of the invasion process that began in the Suez Canal. This seems to validate the hypothesis of Lessepsian immigration, called into question by Bazzicalupo et al. [17] based on molecular analyses.

The establishment phase (1967–2000) marked a significant increase in occurrences, mainly concentrated in the central Mediterranean (as shown in Figure 4c), as well as spatial dispersion. The species started to colonize the western Basin, leading to the shift in the median center, evident both in the establishment and the expansion phase (as shown in Figure 6). At this stage, the first nuclei of aggregation began to emerge.

Expansion (2001–2024) revealed intensified dispersal, with new aggregation nuclei emerging in the western Mediterranean, such as in Spain, and reinforcing in Sicily, the Adriatic, and the Aegean Sea. It has to be highlighted, as the species density in the Aegean intensified in a short time (from 2001 to 2009) compared to the other areas, where similar densities were reached over more than forty years. Furthermore, as confirmed by GOG* analysis, the Aegean Sea emerged as a key area of intense settlement of *B. leachii*. The same consideration can be made for the northern Adriatic Sea, where the species continues to be present, as indicated by the high outlier identified. Both areas are mainly featured by semi-enclosed bays offering species’ preferred habitat in shallow and sheltered water with sandy muddy bottoms, where most sightings occur.

Another nucleus of intensification is the Spanish one, whose kernel density rapidly increased from 2010 to 2017. The trends observed in the Aegean Sea and Spain follow the rapid increase in Mediterranean Sea surface temperatures over the last two decades [38,39] that could have favored the establishment and expansion of *B. leachii*. The aggregation of Spanish occurrences formed an isolated recent hotspot compared to the other nuclei (Figure 4f), likely deriving from an independent income from the Atlantic Ocean.

### 4.1. Likely Scenarios of the Invasion Process

As mentioned before, the most likely hypothesis of *B. leachii* spread is that the species entered the Mediterranean Sea through the Suez Canal before the 1940s. It was subsequently transported, in its larval stage, by the Asia Minor surface current, which flows from the southeastern Mediterranean northward and then enters the Aegean Sea through the straits of the Cretan Arc [40]. According to Paige [14], indeed, ragged sea hare larvae take nineteen days to metamorphose after hatching but are still able to delay settlement for several months until a suitable substrate is identified. The delay period may provide ample time for the larvae to travel long distances, as observed in the Gulf of Mexico and the Caribbean [14].

The next step of expansion, the conquest of the Maltese and Italian waters, is difficult to explain solely through natural diffusion, since the records distribution is discontinuous. Secondary dispersion via anthropogenic means is a likely scenario. Although larval transfer through ballast water seems less likely, since the survival rate of planktonic organisms decreases rapidly already in the first few days of transport [41], short ship routes could allow for larval survival promoting dispersion. In fact, the Maltese record is within a harbor and the several Italian ones are reported in the Mar Grande and Mar Piccolo of Taranto (Ionian Sea), areas characterized by historically intense maritime traffic. A similar scenario may have happened in the Adriatic Sea, where the species was first recorded in the port of Bari (southern Adriatic) and later further north, in the lagoon of Venice and in the Gulf of Trieste, both areas with a high level of maritime traffic.

Another plausible scenario for the arrival of *B. leachii* along the Italian coasts involves aquaculture activities. Monnier et al. [42] put forward the hypothesis that such activities could be the means of the species’ introduction into the lagoons where such activities are carried out, as happens in most of the cited invasion areas. All the above does not exclude the possibility that the species reached the Italian coasts from Aegean Sea unaided, given its high dispersal capability.

The origin of the Spanish nucleus could be different: the species could have crossed the Strait of Gibraltar via Atlantic Ocean currents, first reaching the Balearic Islands and subsequently spreading to the Spanish mainland coast. This hypothesis is supported by the detection of a hotspot in the Spanish coast, as obtained through the GOG* analysis, although a Mediterranean origin cannot be ruled out. In light of the results obtained, *B. leachii* would have invaded the Mediterranean at different times and from multiple fronts—both naturally and through human intervention—and would have settled aided by the progressive warming of surface waters.

### 4.2. Remarks on the Invasiveness Characteristics of B. leachii

*B. leachii* is listed among the 100 worst invasive species in the Mediterranean, having a strong impact on biodiversity [43]. It is capable of living in environments with a wide range of temperature and high salinity values [14,44], preferring shallow waters with low-intensity wave action. Furthermore, its larval plasticity and ability to produce secretions (i.e., purple ink and opaline) that serve as a deterrent to predators and as a warning to potential threats for conspecifics [15,45] make this species an excellent invader. The diet of *B. leachii* is mainly based on benthic cyanobacteria [15] and macroalgae [14]. Its grazing behavior on epiphytic algae could then alter nutrient dynamics in invaded habitats with possible repercussions on local biodiversity, particularly when large aggregations of *B. leachii* are observed during certain periods of the year (autumn and winter, according to Russo [46]). However, while its impact on biodiversity in the Mediterranean Sea has yet to be fully demonstrated, its pharmacological potential is well known, since anticancer, antitumour, and antiviral compounds can be extracted from the species [47,48]. To date, the species is of great interest in numerous experimental studies for further medical uses but also for potential biotechnological applications in different fields [49,50,51].

The potential for negative impacts on native communities emphasizes the need to monitor the distribution of *B. leachii*, particularly in or near protected or sensitive areas. The report of the species in the new location in the Strait of Sicily, i.e., the island of Lampedusa mentioned in the present paper, deserves attention, since the island hosts a Marine Protected Area and offers the preferred habitat of *B. leachii* in its southern and eastern parts where there are several small sheltered bays in shallow waters. Given the low number of observations, its establishment with a viable population on the island of Lampedusa is not confirmed, although its detection validates the Straits of Sicily as a key area of intense settlement of the species, as detected by the aggregation pattern analysis.

## 5. Conclusions

What is the point of tracing the mode of entry of a species into an area where it was previously absent? The comprehension of whether a species is alien or cryptogenic allows for more informed conservation management efforts. In case a cryptogenic species turns out to be invasive, a different approach may be needed than for a non-indigenous one as, for example, is addressed in some European and Mediterranean directives. Although many experts tend to consider *B. leachii* as cryptogenic in the Mediterranean Sea, since its origin is considered uncertain [1], in our opinion, it would be a mistake to pigeonhole the species into a single category for the entire Basin. Our results instead suggest categorizing it as alien at least for some areas where doubt about the origin of the species is less, that is, Maltese waters, Italian Ionian, and the Italian Adriatic Sea.

Our findings provide valuable insights into the invasion dynamics of this species in the Mediterranean Sea and highlight the need for continuous monitoring and management strategies to control the population.

## Figures and Tables

**Table 1:** Analyses, spatial and temporal indicators, and their ecological meaning, including methods and spatial and time scales used (modified from Perzia et al. [18]). Only the first records within a 0.05° lat/long grid were considered for analyses.

Analysis/ Indicator Name	Tools	Spatial Scale	Time Scale	Ecological Meaning
Temporal and spatial–temporal pattern				
Occurrence increase	Cumulative curve of occurrence	Global	All years	Identification of invasion phases; progression of the invasion
Occurrence increasing rate	Evaluation of the slopes of the cumulative curve by least squares method	Global	1933–1966 1967–2000 2001–2024	The rate of specimens increasing in time
Density hotspots	Kernel density Distance radius = 313 km	Global	1933–1966 1933–1983 1933–2000 1933–2009 1933–2017 1933–2024	Expansion areas; nuclei of record aggregation; occurrence of persistent areas; space–time occurrence density increase; highest density areas
Aggregation patterns and spatial structure				
Spatial autocorrelation for a series of increasing distances	Incremental spatial autocorrelation (ISA) (based on Global Moran’s I) Number of distance bands = 10	Global	All years	The distances where the clustering spatial processes are most pronounced. Distribution pattern: dispersion vs. random vs. clustering. Change in the spatial pattern over time
Statistically significant hot spots and cold spots	Getis–Ord Gi* (GOG*) Hot spot analysis Distance band = 313 km	Local	All years	Initial and current direction of spread and identification of dispersion/settle areas
Spatial outliers	Anselin local Moran’s I (AMI) Outlier analysis Distance band = 313 km	Local	All years	Recent records in proximity of a group of older records and vice versa
Key characteristics of distribution				
Center of gravity	Central tendency (median center)	Global	1933–1966 1933–2000 1933–2024	Species concentration center and its change over time
Directional Dispersion	XStdDist, YStdDist; (km) Standard deviational ellipse (1 standard deviation)	Global		Species distribution in X and Y directions
Directional trends	Rotation (°) Standard deviational ellipse (1 standard deviation)	Global		Directional trend of species dispersion

**Table 2:** Key characteristics of the distribution of *B. leachii* records in the Mediterranean Sea, calculated for the periods 1933–1963, 1967–2000, and 2001–2024. Metrics include central tendency (as median center), directional dispersion, and directional trends. DD = decimal degrees.

Indicator	Method	Unit	1933–1963	1967–2000	2001–2024
Central tendency	Median center	Longitude (DD) Latitude (DD)	33.52 32.91	16.17 40.03	16.58 39.80
Directional dispersion	Standard deviational ellipse	XStdDist (km) YStdDist (km)	866 182	1184 479	1256 425
Directional trends	Standard deviational ellipse	Rotation (°)	123	104	94

## Data Availability

Dataset available upon request from the authors.
